# Tunable Coupled-Resonator-Induced Transparency in a Photonic Crystal System Based on a Multilayer-Insulator Graphene Stack

**DOI:** 10.3390/ma11102042

**Published:** 2018-10-19

**Authors:** Hanqing Liu, Jianfeng Tan, Peiguo Liu, Li-an Bian, Song Zha

**Affiliations:** 1 College of Electronic Science and Engineering, National University of Defense Technology, Changsha 410073, China; 18674895435@163.com (H.L.); pg731@126.com (P.L.); zhasong0731@126.com (S.Z.); 2 School of Physical and Electronic Science, Changsha University of Science and Technology, Changsha 410114, China; yimuhengdi@163.com

**Keywords:** coupled-resonant-induced transparency, photonic crystal, graphene, chemical potential

## Abstract

We achieve the effective modulation of coupled-resonator-induced transparency (CRIT) in a photonic crystal system which consists of photonic crystal waveguide (PCW), defect cavities, and a multilayer graphene-insulator stack (MGIS). Simulation results show that the wavelength of transparency window can be effectively tuned through varying the chemical potential of graphene in MGIS. The peak value of the CRIT effect is closely related to the structural parameters of our proposed system. Tunable Multipeak CRIT is also realized in the four-resonator-coupled photonic crystal system by modulating the chemical potentials of MGISs in different cavity units. This system paves a novel way toward multichannel-selective filters, optical sensors, and nonlinear devices.

## 1. Introduction

The electromagnetically induced transparency (EIT) effect, which is a quantum interference phenomenon that forms a sharp transparency window over a broad transmission spectrum, has a wide range of potential applications in the fields of slow optical propagation, the transfer of quantum correlation, and nonlinear optical processes [1,2,3]. EIT-like optical responses can be obtained in classical resonator systems and many optical devices, such as grating and plasmonic resonator antennas, which has opened up a pathway in photonics, offering potential for smaller devices to be used for the manipulation and transmission of light [4,5]. The EIT-like effect discovered in the coupled-resonator photonic systems is also called coupled-resonator-induced transparency (CRIT). In previous studies, it was theoretically predicted and experimentally demonstrated that the CRIT effect can efficiently provide tunable transparency on the optical chip due to the significant reduction in the threshold power for optical bistability [6,7,8,9,10]. However, varying the optical dispersion relations, symmetries, and spatial distribution once the physical parameters of the system are fixed is difficult, which means the tunability of CRIT would be limited after the fabrication of the whole resonant system.

Since its discovery in 2004, graphene, a monolayer of carbon atoms arranged in a two-dimensional (2D) hexagonal lattice, has received considerable attention due to its electrical and thermal properties, as well as unique atomic thickness [11,12]. An undoped graphene monolayer absorbs approximately 2.3% of normal incident light across the visible and infrared range, which is high given its single atom thickness [13,14]. The conductivity of graphene can be tuned under electric/magnetic biasing or chemical doping; thus, a variety of graphene-based structures have been proposed to achieve many tunable optical responses, such as propagation in optical waveguides, perfect absorption in photonic crystal arrays, resonance on metamaterials, and nonlinear phenomena [15,16]. However, graphene is generally thought to not conduct sufficiently well to replace metals in optical devices, since it is too “thin” to sustain intense resonance [17,18]. To overcome this limitation, multiple graphene structures have been proposed to replace the monolayer graphene due to the enlarged amplitude of the graphene-light interaction. For example, Zhu et al. [19] designed a terahertz fishnet metamaterial consisting of a gallium arsenide substrate sandwiched between multi-layer graphene-dielectric composites, which dynamically controlled the left-handed features by applying external bias to shift the Fermi level in graphene. Su et al. [20] proposed an ultra-thin terahertz metamaterial absorber based on graphene/MgF_2_ multilayer stacking unit cells arrayed on a gold (Au)-film plane and obtained two total absorption peaks for the incident light in the terahertz range.

In the present study, we design a novel coupled-resonator photonic crystal system, which is composed of a photonic crystal waveguide (PCW) side coupled with two kinds of resonant cavities: one embedded with a multilayer graphene-insulator stack (MGIS) directly coupled to the PCW, and a defect cavity coupled to the first one. The light-graphene interaction in our proposed structure can be significantly enhanced due to the combination of multiple-layer graphene stacks and resonance defect cavities. The transmission spectrum of our proposed system exhibits a transparency window over the resonant band due to the interference between the optical pathways in the two cavities, which reveals a classical CRIT effect. The peak frequency of CRIT can be adjusted by varying the chemical potential of graphene. Furthermore, we design a four-resonator-coupled photonic crystal system and obtain a tunable multipeak CRIT effect.

## 2. Theoretical Modeling

**Figure 1:**
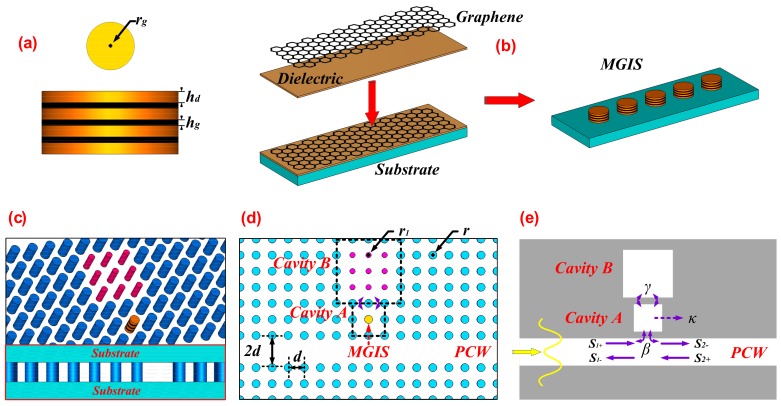
(**a**) The top view and side view of a multilayer graphene-insulator stack (MGIS) and (**b**) the modeling process of MGIS. (**c**) Schematic perspective view (the bottom illustration shows the side view), (**d**) top view, and (**e**) propagating model of the proposed photonic crystal system.

The top view and side view of MGIS are schematically visualized in Figure 1a. Monolayer graphene with a thickness of *h_g_* = 0.34 nm is separated by dielectric layers [21]. Here, we chose Al_2_O_3_ as the insulating dielectric with a relative permittivity of $\varepsilon_{d}=4.9$, which is also a universal material used in supporting the multiple graphene structures [10,22]. Distributing the carriers into multiple stacks effectively enhances the magnitude of resonance compared with doping in single-layer graphene, which can promote the light-graphene interaction [23]. The thickness $h_{d}$ of the Al_2_O_3_ layer should be a deep subwavelength thickness, but thick enough to avoid the interaction between adjacent graphene layers (e.g., interlayer transitions) [24], thus we define $h_{d}=50\mathrm{nm}$. In addition, the radius of MGIS is set as $r_{g}=45\mathrm{nm}$. The MGIS can be homogenized and viewed as an anisotropic uniaxial medium using the effective medium approximation, and its effective out-of-plane and in-plane permittivities are derived by taking the long-wavelength limit of the Bloch theory [25]:

(1) $$\begin{array}{c} \varepsilon_{\left|\right|}=\varepsilon_{d}+\frac{i\sigma_{g}\left(\omega \right)}{\omega \varepsilon_{0}h_{d}}\\ \varepsilon_{⊥}=\varepsilon_{d} \end{array}$$

where $\varepsilon_{\left|\right|}$ and $\varepsilon_{⊥}$ are the permittivity parallel and perpendicular to the horizontal plane, respectively; ω represents the angular frequency; and $\sigma_{g}$ is the surface conductivity of monolayer graphene, which contains two contributed portions: $\sigma_{\mathrm{intra}}\left(\omega \right)$ represents absorption due to intraband electron-photon scattering, and $\sigma_{\mathrm{inter}}\left(\omega \right)$ is caused by the interband electron transition process. Their expressions are given by the Kubo formula [26]:

(2) $$\begin{array}{c} \sigma_{\mathrm{intra}}\left(\omega \right)=j\frac{q^{2}}{\pi \hbar \left(\hbar \omega +j\Gamma_{c}\right)}\left[\mu_{c}+2k_{B}T\ln\left(e^{-\mu_{c}/k_{B}T}+1\right)\right]\\ \sigma_{\mathrm{inter}}\left(\omega \right)=j\frac{q^{2}}{4\pi \hbar }\ln\left[\frac{2\left|\mu_{c}\right|-\left(\hbar \omega +j\Gamma_{c}\right)}{2\left|\mu_{c}\right|+\left(\hbar \omega +j\Gamma_{c}\right)}\right] \end{array}$$

where q is the charge of the electron, *T* is the temperature, ℏ is the reduced Planck constant, $k_{B}$ is the Boltzmann constant, and $\mu_{c}$ is the chemical potential of graphene. $\Gamma_{c}$ represents the damping constant, which can be defined as $\Gamma_{c}=q\hbar \nu_{f}^{2}/\mu \mu_{c}$, where $\nu_{f}$ is the Fermi velocity and μ is the electron mobility [19]. From Equations (1) and (2), the value of $\sigma_{g}$ is closely connected with the chemical potential, which can be modulated by chemical doping methods or applying gate voltage [27,28].

Composed of periodical silicon medium elements, 2D photonic crystal systems have potential for optical communication given their potential ability to control the propagation of light [29,30]. As shown in Figure 1c, our proposed photonic crystal system mainly consisted of a photonic crystal waveguide (PCW), a directly side-coupled cavity (*Cavity A*), and a defect cavity (*Cavity B*). The PCW was formed by removing a specific row of silicon elements, which realize guiding and trapping the transverse-magnetic (TM) light in the remaining empty space as light injected and coupled into the photonic crystal system [31]. The bottom illustration in Figure 1c is the side view of our proposed system. Since the system is fabricated by a periodic silicon rods array, both the top and bottom surface are loaded with silicon substrate to support the whole structure and guarantee the normal propagation of the incident wave in PCW. Figure 1d shows the top view of the proposed system. We defined the lattice constant d of our proposed system as 400 nm, the width of the excitation port of PCW as 2d, and the radius r of the silicon element as 90 nm. The height of the proposed system was set as 251.4 nm. *Cavity A*, which was fabricated by embedding the MGIS into the resonant cavity to replace the original silicon element of the structure, was located at distance *2d* from the center of the PCW. The radius of the MGIS in *Cavity A* was equal to $r_{g}$, as shown in Figure 1a. The defect cavity *Cavity B* was formed by a 3 × 3 silicon element array with a periodic radius of $r_{1}=40\mathrm{nm}$. Although the tunable CRIT effect in our paper was only studied in theory and simulated on software, the modeling process of MGIS has already been presented experimentally in previous studies [10,11]. The first step of the process, presented in Figure 1b, is coating the monolayer graphene and dielectric layer by turns to obtain an unpatterned MGIS on the substrate; then, the next step is patterning the unpatterned MGIS into the desirable shape, such as a cylinder; the third step is reserving only one MGIS on the substrate and eliminating the others; and the last step is coating silicon on the substrate and patterning the silicon layer to the photonic crystal system. In the whole of the fabricating process, a great challenge is how to keep the MGIS unpatterned and stable in the last step.

As the incident wave passed through the coupled aperture, the energy was coupled into *Cavity A* through the dielectric aperture and excited the resonant modes at special wavelengths. Here, the temporal coupled-mode theory can be used to analyze the dynamic transmission characteristics of our proposed system. As shown in Figure 1e, β denotes the coupling coefficient between PCW and *Cavity A*, and κ is the decay rate due to the internal loss in *Cavity A,* which is mainly caused by the photonic crystal structure itself. γ denotes the coupling coefficient between *Cavity A* and *Cavity B*. Since the light-graphene interaction in MGIS can absorb considerable injected energy in *Cavity A*, the value of γ is closely related to the absorption performance of MGIS. $S_{1+}$, $S_{1-}$, and $S_{2-}$ represent the input, reflection, and output field amplitude, respectively, and the differential relationships between the parameters above can be expressed as [32]:

(3) $$\frac{da}{dt}=\left(-j\omega_{1}-\beta -\gamma -\kappa \right)a+j\sqrt{\beta }S_{1+}+j\sqrt{\gamma }b$$

(4) $$\frac{db}{dt}=-j\omega_{2}b-\gamma b+j\sqrt{\gamma }a$$

where a and b denote the normalized amplitude of *Cavity A* and *Cavity B*, respectively; $\omega_{1}$ and $\omega_{2}$ represent the resonant frequency of *Cavity A* and *Cavity B*, respectively. Given the power conservation and the time reversal symmetry, the relationship among the amplitudes in PCW can be calculated as: $S_{2-}=S_{1+}+j\sqrt{\beta }a$ and $S_{1-}=j\sqrt{\beta }a$. In the linear system, since the field everywhere oscillates and the input frequency is ω, we have da/dt=−jωa and db/dt=−jωb. When the input field is launched to the left port ($S_{2+}=0$), the transmission coefficient t of the whole system is derived as:

(5) $$t=\frac{S_{2-}}{S_{1+}}=1-\beta /\left(\left[j\left(\omega_{1}-\omega \right)+\left(\beta +\gamma +\kappa \right)\right]+\frac{\gamma }{\left[j\left(\omega_{2}-\omega \right)+\gamma \right]}\right)$$

Particularly, without *Cavity B*, the transmission coefficient can be simplified as:

(6) $$t=1-\beta /\left[j\left(\omega_{1}-\omega \right)+\beta +\kappa \right]$$

**Figure 2:**
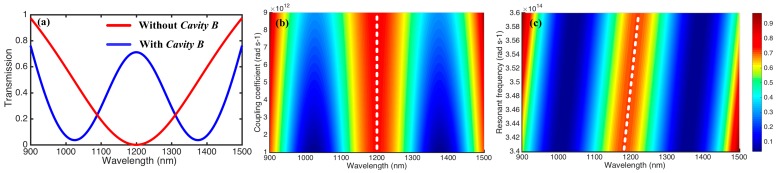
(**a**) Transmission spectrum of the proposed system calculated by the temporal coupled-mode theory. (**b**) Evolution of transmission spectrum with the coupling coefficient γ varying from 1 × 10^12^ rad·s^−1^ to 9 × 10^12^ rad·s^−1^ when the other parameters are fixed. (**c**) Evolution of transmission spectrum with the resonant frequency $\omega_{1}$ of *Cavity A* varying from 3.4 × 10^14^ rad·s^−1^ to 3.6 × 10^14^ rad·s^−1^ when the other parameters are fixed.

We calculated the transmission response to verify the CRIT effect of our proposed system when the parameters in Equations (5) and (6) were set to $\omega_{1}=3.5\times 10^{14}\mathrm{rad}\cdot s^{-1}$, $\omega_{2}=2.7\times 10^{14}\mathrm{rad}\cdot s^{-1}$, $\kappa =1.5\times 10^{11}\mathrm{rad}\cdot s^{-1}$, $\beta =4.3\times 10^{14}\mathrm{rad}\cdot s^{-1}$, and $\gamma =1.2\times 10^{12}\mathrm{rad}\cdot s^{-1}$. Figure 2a shows the transmission spectrum as the incident wave varies from 900 to 1500 nm, both with and without *Cavity B*. When *Cavity A* was not side-coupled with *Cavity B*, the value of γ was zero, and the spectrum exhibited a dip at the resonance wavelength due to the destructive interference between the incident wave and the escaped power from *Cavity A*. However, when *Cavity A* was side-coupled with *Cavity B*, a narrow transparency window appeared in the center of a broader transmitted dip, which showed an EIT-like phenomenon. This performance is also known as CRIT, similar to EIT in the atomic system, which is derived from the destructive interference between the two optical pathways passing and bypassing *Cavity B*. For Equation (5), under the condition of $\omega \approx \omega_{1}\approx \omega_{2}$, the value of t can be simplified as 1−β/(β+γ+κ+1), which means an obvious transparency window. Figure 2b,c reveal the evolution of the transmission spectrum with the values of γ and $\omega_{1}$, respectively. The results show that the peak value of the transparency window gradually increased as γ increased from 1 × 10^12^ rad·s^−1^ to 9 × 10^12^ rad·s^−1^, whereas the frequency corresponding to the transparency window processed a nearly linear blue shift as $\omega_{1}$ increased from 3.4 × 10^14^ rad·s^−1^ to 3.6 × 10^14^ rad·s^−1^. The CRIT effect can be used to design bandpass plasmonic filters, thus the coupling coefficients and resonant frequencies of the cavities enable control of the filtering features, such as wavelength and bandwidth [33].

## 3. Simulation Results

**Figure 3:**
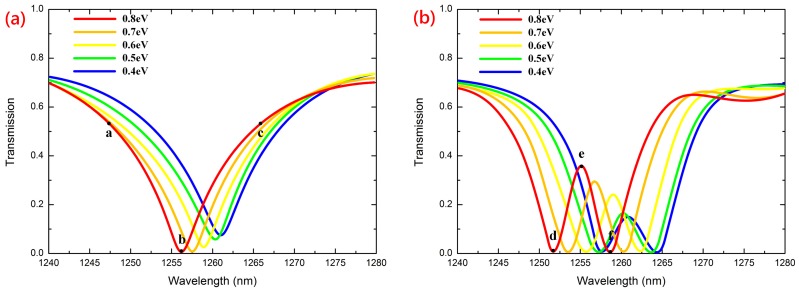
Simulation results of transmission spectrum of our proposed photonic crystal system as the chemical potential of MGIS varies from 0.8 eV to 0.4 eV, in (**a**) without *Cavity B* and (**b**) with *Cavity B*.

The transmission response of the proposed photonic crystal system was investigated numerically using the rigorous finite element method implemented on software COMSOL. Particularly, we define the scattering boundary condition on the top and bottom surfaces, which can effectively eliminate the losses caused by environments such as substrates. For the permittivity parameters of MGIS mentioned in Equations (1) and (2), at room temperature, we assumed that the chemical potential $\mu_{c}$ was 0.8 eV, the Fermi velocity $\nu_{f}$ was *c*/300 m/s, and the electron mobility μ was 10,000 cm^2^/Vs. We first simulated the transmission spectrum of our system without *Cavity B*. Figure 3a (red line) shows that there is a sharp resonant band over the wavelength range from 1240.0 nm to 1280.0 nm, and the transmission coefficient nearly reduced to zero at about 1256.2 nm. In Figure 3b (red line), when *Cavity A* is coupled with *Cavity B*, a transparency window is formed in the resonant stop band and the transmission coefficient can reach 0.36 at about 1255.1 nm, which denotes a typical CRIT phenomenon. The results are in good agreement with the theoretical modeling in Figure 2a. Since the permittivity of MGIS can be effectively modulated by tuning the chemical potential $\mu_{c}$ of graphene by applying gate voltage, we also discussed the transmission spectrum with different values of $\mu_{c}$. In Figure 3a, the wavelength corresponding to the resonance gradually increases from 1256.2 nm to 1261.8 nm as $\mu_{c}$ decreases from 0.8 eV to 0.4 eV. In Figure 3b, the wavelength corresponding to the transparency window displays a red shift to 1260.9 nm and the peak value gradually decreases from 0.36 to 0.17.

**Figure 4:**
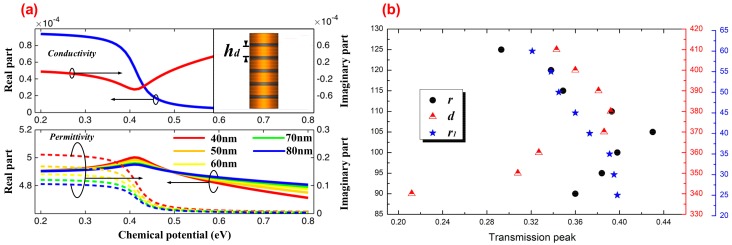
(**a**) At a wavelength of 1250 nm, the conductivity of the monolayer graphene (blue line represents real part and red line represents imaginary part) and the in-plane permittivity of MGIS with different values of $h_{d}$ (solid line represents real part and dotted line represents imaginary part) as a function of $\mu_{c}$. (**b**) The variation trend in the transmission peak when $\mu_{c}=0.8\mathrm{eV}$ with different structural parameters.

Here, we introduce the dielectric resonator perturbation theory to explain the variation in wavelength corresponding to the resonant band and transparency window along with the chemical potential in Figure 3. According to Equations (1) and (2), we first calculated the value of $\sigma_{g}$ and $\varepsilon_{\left|\right|}$ at about 1250 nm, corresponding to the photon energy ℏω=0.994eV, as shown in Figure 4a. For the conductivity $\sigma_{g}$ of the monolayer graphene, the real part (blue line) reduced rapidly when $\mu_{c}$ was in the region of 0.3 to 0.5 eV, but changed slowly in other regions, while the imaginary part (red line) first decreased and then increased as $\mu_{c}$ increased. Thus, we observed that graphene’s conductivity is strongly dispersive and extremely sensitive to the bias Fermi level, which immediately suggests the potential for graphene’s usage in tunable optical metamaterials and metadevices. For the permittivity $\varepsilon_{\left|\right|}$ of MGIS, in contrast to the variation trend of $\sigma_{g}$, the real part of $\varepsilon_{\left|\right|}$ (solid line) first increased and then decreased, while the imaginary part (dashed line) reduced rapidly in the region of 0.3 to 0.5 eV, but changed slowly in other regions. Particularly, the modulation amplitude of $\varepsilon_{\left|\right|}$ significantly enhanced as $h_{d}$ decreased from 80 nm to 40 nm, which means we can improve the tunability of $\varepsilon_{\left|\right|}$ by moderately reducing the thickness of the dielectric layer. Since we only tuned the permittivity of MGIS with $\mu_{c}$ relying on the applied voltage and maintained the volume of *Cavity A*, according to the perturbation theory, the modulation for the resonant frequency of *Cavity A* under the condition without volume dilatation can be expressed as:

(7) $$\frac{\Delta \lambda }{\lambda_{0}}=\frac{\int_{\Delta V}\left(\Delta \varepsilon {\left|E_{0}\right|}^{2}+\Delta \mu {\left|H_{0}\right|}^{2}\right)dV}{\int_{\Delta V}\left(\varepsilon_{0}{\left|E_{0}\right|}^{2}+\mu_{0}{\left|H_{0}\right|}^{2}\right)dV}$$

where $E_{0}$ and $H_{0}$ represent the E-field intensity and H-field intensity in *Cavity A* before perturbation, respectively;$\lambda_{0}$, $\varepsilon_{0}$, and $\mu_{0}$ are the resonant wavelength, permittivity, and permeability of *Cavity A* before perturbation, respectively; and Δλ, Δε, and Δμ are the corresponding variations after perturbation. In the case of Δε>0,Δλ>0. The permittivity of MGIS displayed a negative relationship with chemical potential $\mu_{c}$ in the region of 0.4 to 0.8 eV (Figure 4a), which means the resonant wavelength of *Cavity A* decreases as $\mu_{c}$ increases. The wavelength of the transparency window is positively related to resonant wavelength (Figure 2c). Thus, with analysis combined with the perturbation theory, we drew the conclusion that as $\mu_{c}$ increases from 0.4 eV to 0.8 eV, the wavelength of the transparency window shows a blue shift, which perfectly matches the simulation results, as shown in Figure 3b.

**Figure 5:**
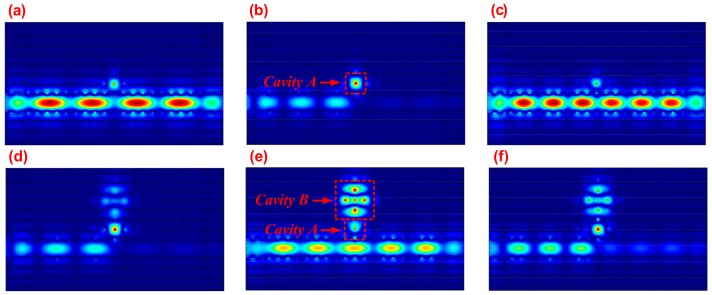
In case of $\mu_{c}=0.8\mathrm{eV}$, the power flow distribution of our proposed photonic crystal system without *Cavity B* at about (**a**) 1247.1 nm, (**b**) 1256.2 nm, and (**c**) 1265.3 nm, and with *Cavity B* at about (**d**) 1252.3 nm, (**e**) 1255.1 nm, and (**f**) 1258.2 nm.

From the simulation above, the performance of the CRIT effect was verified to be closely related to the chemical potential of MGIS. The performance can be effectively changed by tuning the structural parameters of the proposed photonic crystal system. Here, we mainly discuss the value of the transparency peak with different parameters, including the radius r of the silicon element, the lattice constant d, and the radius $r_{1}$ of the elements in *Cavity B*. As shown in Figure 4b, defining the chemical potential of graphene at about 0.8 eV, the transparency peak first increased and then declined as *r* increased from 90 to 125 nm, and reached 0.43 when r was equal to 105 nm. A similar trend also occurred as *d* increased from 340 to 410 nm. These variations can be explained by the appropriate reduction in the lattice constant or increase in the radius of the element alleviating the propagation loss and leading to an improvement of transmission [34,35]. However, a too compact configuration of elements can destroy the periodic property of the whole photonic crystal system and hold the incident wave back to the defect cavity. The decrease in $r_{1}$ can affect the resonant property of *Cavity B*, resulting in a gradual increase in the transparency window from 0.32 to 0.4. In order to better understand the physical mechanism of the CRIT effect, we also plotted the power flow distributions when the value of the chemical potential was set as 0.8 eV, which reflected the transmission path of the incident light wave in our proposed system. Figure 5a–c show the power flow distributions corresponding to the dots “a”, “b”, and “c” in Figure 3a, respectively. Consistent with the variation trend in the transmission spectrum, the incident waves can pass through the PCW smoothly at about 1247.1 nm and 1265.3 nm; conversely, the propagation dissipates rapidly and most of the power is concentrated into *Cavity A* at about 1256.2 nm. These results verify that the resonance band is due to the absorption of MGIS caused by the enhanced graphene-light interaction. Figure 5d–f show the power flow distributions corresponding to the dots “d”, “e”, and “f” in Figure 3b, respectively. There is coherence enhancement between the electric fields in *Cavity A* and in *Cavity B* at about 1255.1 nm, whereas the incident light in PCW is in-phase with the wave in *Cavity A*. Thus, the CRIT effect can also be explained by the interference between the two optical pathways: the direct excitation of resonant mode in *Cavity A* by the incident wave and the excitation by coupling with *Cavity B* [36]. The variation in the chemical potential of graphene in MGIS directly affects the interference between the PCW and resonant cavities to change the CRIT effect. In addition, at about 1252.3 nm and 1258.2 nm, there is an anti-phase of the wave between *Cavity A* and *Cavity B*, which means the conditions of resonance destruction are perfectly satisfied and lead to the cut-off of propagation.

## 4. Discussion

### 4.1. Multipeak CRIT Effect

**Figure 6:**
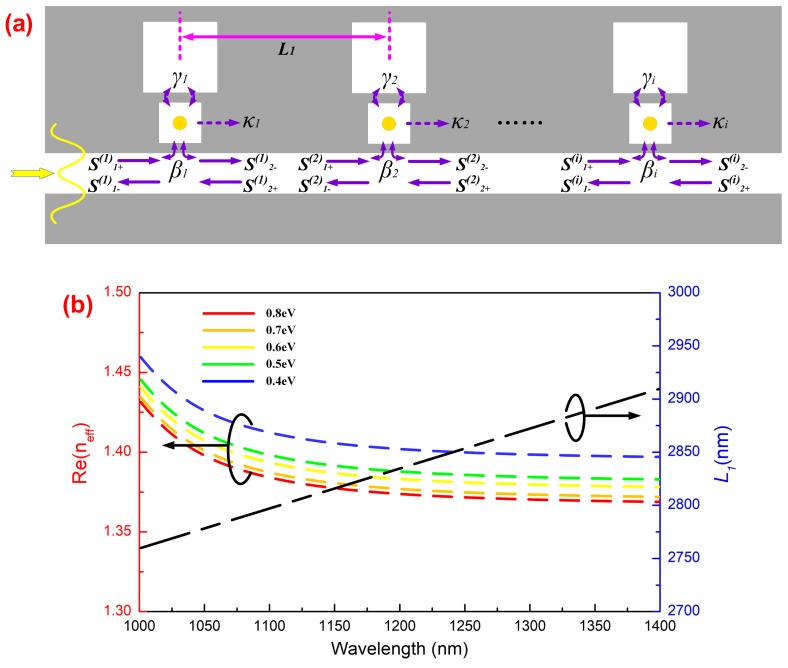
(**a**) Propagation model of the multi-nanoresonator-coupled photonic crystal systems. (**b**) Real part of the effective refractive index in the PCW with $\mu_{c}=0.8\mathrm{eV}$ and the approximately optimal separation $L_{1}$ corresponds to the adjacent cavity units phase equal to π for the central wavelength.

For the multi-resonator-coupled photonic crystal system in Figure 6a, the dynamic transmission features can be investigated using temporal coupled-mode theory. The transmission coefficient $t_{i}$ of incident light passing through the *i*th side-coupled-cavity unit is:

(8) $$t_{i}=\frac{S_{2-}^{\left(i\right)}}{S_{1+}^{\left(i\right)}}=1-\beta_{i}/\left(\left[j\left(\omega_{1,i}-\omega \right)+\left(\beta_{i}+\gamma_{i}+\kappa_{i}\right)\right]+\frac{\gamma_{i}}{\left[j\left(\omega_{2,i}-\omega \right)+\gamma_{i}\right]}\right)$$

where $\beta_{i}$, $\gamma_{i}$, and $\kappa_{i}$ represent the decay rates in the *i*th cavity unit; and $\omega_{1,i}$ and $\omega_{2,i}$ are the resonant frequency of cavities. The propagation waves in the PCW should satisfy the relationship in the steady state:

(9) $$S_{2+}^{\left(i\right)}=S_{1-}^{\left(i+1\right)}e^{j\varphi_{i}},S_{1+}^{\left(i+1\right)}=S_{2-}^{\left(i\right)}e^{j\varphi_{i}}$$

where $\varphi_{i}$ represents the phase difference between the *i*th and *i + 1*th cavity unit, which can be expressed as [37,38]:

(10) φi=ωRe(neff,i)Lic+θ

(11) $$\varepsilon_{\left|\right|}\sqrt{n^{2}{}_{eff,i}-\varepsilon_{0}}\tanh\left(\frac{2d\omega \pi \sqrt{n^{2}{}_{eff,i}-\varepsilon_{0}}}{c}\right)+\varepsilon_{0}\sqrt{n^{2}{}_{eff,i}-\varepsilon_{\left|\right|}}=0$$

where $L_{i}$ is the separation distance between the *i*th and *i + 1*th cavity units, c is the light velocity in a vacuum, $n_{eff,i}$ is the effective refractive index of the fundamental mode in the PCW, and $\varepsilon_{0}$ and $\varepsilon_{\left|\right|}$ are the permittivity of vacuum and the MGIS in *Cavity A*, respectively. θ is the additional phase shift of *Cavity B*. Assume that the parameters of the whole system are equal to the values defined in Figure 3. The real part of the refractive index Re(neff,1) as a function of incident wavelength calculated by Equation (11) is shown in Figure 6b. When $\mu_{c}=0.8\mathrm{eV}$, the value of Re(neff,1) gradually decreases from 1.472 to 1.401 as the wave frequency increases from 1000 nm to 1400 nm. Since the permittivity of graphene is modulated with the chemical potential, we found that the integral level of the refractive index declined as $\mu_{c}$ reduced to 0.4 eV. In addition, Figure 6b shows the value of $L_{1}$ as a function of incident wavelength (black dotted line) when $\mu_{c}=0.8\mathrm{eV}$, which makes the value of $\varphi_{1}$ satisfy the odd times of *π*. In the region of λ≥1150nm, the variation in $L_{1}$ tends to be stable. As a result, according to Equations (10) and (11), to realize a multipeak CRIT effect in our proposed system, both the distance $L_{1}$ between adjacent cavity units and the resonant property of each cavity are important factors.

**Figure 7:**
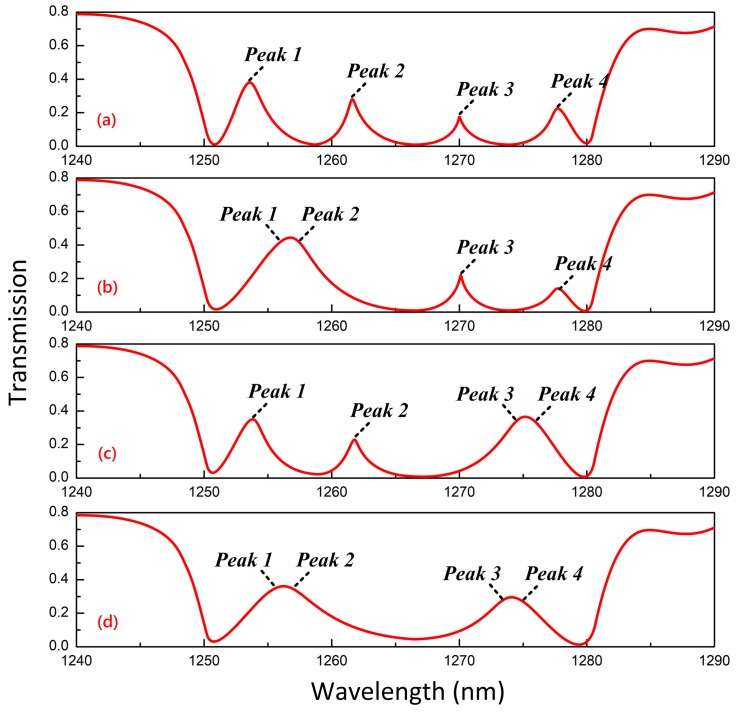
Transmission spectrum of our proposed four-resonator-coupled photonic crystal system when: (**a**) $\mu_{1}=\mu_{2}=\mu_{3}=\mu_{4}=0.8\mathrm{eV}$; (**b**) $\mu_{1}=0.68\mathrm{eV}$, $\mu_{2}=0.89\mathrm{eV}$, and $\mu_{3}=\mu_{4}=0.8\mathrm{eV}$; (**c**) $\mu_{1}=\mu_{2}=0.8\mathrm{eV}$, $\mu_{3}=0.55\mathrm{eV}$, and $\mu_{4}=0.93\mathrm{eV}$; and (**d**) $\mu_{1}=0.68\mathrm{eV}$, $\mu_{2}=0.89\mathrm{eV}$, $\mu_{3}=0.55\mathrm{eV}$, and $\mu_{4}=0.93\mathrm{eV}$.

A four-resonator-coupled photonic crystal system was designed as an example to investigate the multipeak CRIT effect. The separations of adjacent cavity units ($L_{1}$, $L_{2}$, and $L_{3}$) were set to 2800 nm uniformly, which is precisely seven times the lattice constant d. The wavelength of the transparency window in Figure 3 could be tuned from 1255.1 nm to 1260.9 nm by tuning the chemical potential of graphene from 0.8 eV to 0.4 eV. However, this modulation was far from the satisfactory range to achieve the multi-wavelength CRIT effect. An effective method to broaden the modulated range of resonance is changing the radius of the unit in the defect cavity (the unit here is MGIS), which was a common method used to realize the multi-mode effect in prior studies [39,40]. As a result, the radius $r_{g}$ of MGIS in our proposed structure was set to 45, 47, 49, and 51 nm,. Assume that the chemical potentials of graphene in different cavity units are defined as $\mu_{1}$, $\mu_{2}$, $\mu_{3}$, and $\mu_{4}$. Figure 7a reveals the transmission spectrum over the wavelength range from 1240 nm to 1290 nm on the condition that $\mu_{1}=\mu_{2}=\mu_{3}=\mu_{4}=0.8\mathrm{eV}$. In this case, there are four transparency peaks (*peak 1* to *peak 4*) located at 1256.4, 1262.1, 1269.9, and 1277.5 nm, respectively, with peak values of about 0.38, 0.31, 0.19, and 0.23, respectively. The difference in peak values can be explained by two reasons: the difference in the coupling effect between *Cavity A* and *Cavity B* at different wavelengths and the interference between adjacent cavity units. To further investigate the tunability of our proposed system with MGIS, we tuned the value of $\mu_{1}$ to 0.68 eV and the value of $\mu_{2}$ to 0.89 eV and found that *peak 1* and *peak 2* combined into one peak at about 1257.1 nm with a peak value of 0.42, as seen in Figure 7b. This can be explained by the shift in the transparency window along with the chemical potential according to the perturbation theory in Equation (7). Figure 7c illustrates the amalgamation of *peaks 3* and *4* at about 1275.2 nm when the values of $\mu_{3}$ and $\mu_{4}$ were tuned to 0.55 and 0.93 eV, respectively. When further tuning the chemical potential of the MGIS in these four cavity units simultaneously, as shown in Figure 7d, the four transparency peaks in Figure 7a simplified into only two peaks with smaller Q-factors. The modulation property of the multipeak CRIT effect in a multiple cavities system based on MGIS has meaningful applications in terms of broadening the sidelobes of filters and tunable multichannel photonic crystal waveguide.

### 4.2. Electrical Modulation of MGIS

**Figure 8:**
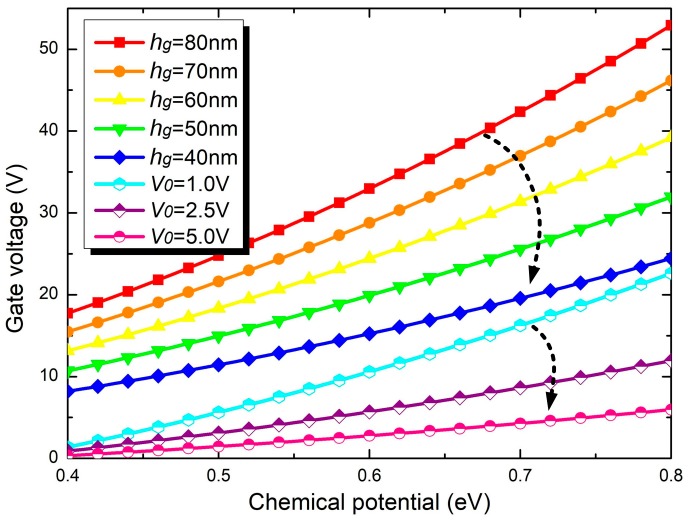
The relationship between $\mu_{c}$ and $V_{g}$ for different thicknesses of the Al_2_O_3_ layer considering the offset voltage $V_{0}$ caused by doping on graphene.

The voltage required to effectively tune the chemical potential of graphene is the key to achieving tunability of the proposed CRIT effect. Here, the relationship between the chemical potential of graphene and applied voltage $V_{g}$ can be denoted by [41]:

(12) $$\mu_{c}=\hbar \nu_{F}\sqrt{\pi \left(n_{S}+\frac{C\left|V_{g}\right|}{q}\right)}$$

where $\nu_{F}$ (1 × 10^6^ m/s) is the Fermi velocity for graphene and C (~24 mF/m^2^) is the effective capacitance per unit area. The intrinsic carrier concentration $n_{S}$ is a constant for monolayer graphene, which is about 1.17 × 10^17^ m^−3^. As graphene is supported on the top of an Al_2_O_3_ dielectric, $n_{S}$ can be expressed as [42]:

(13) $$n_{S}=\frac{\varepsilon_{0}\varepsilon_{d}}{qh_{d}}\left(V_{g}+V_{0}\right)$$

where $\varepsilon_{0}$ (~8.854 × 10^−12^ F/m) denotes the permittivity of the vacuum and $V_{0}$ denotes the offset voltage caused by doping, which can be obtained from Equations (12) and (13) as $V_{g}$ varies with the thickness $h_{d}$ of Al_2_O_3_, as well as the offset voltage $V_{0}$. As shown in Figure 8, assuming that $V_{0}$ is equal to zero, the required $V_{g}$ to tune $\mu_{c}$ increasing from 0.4 eV to 0.8 eV decreases from 52.9 V to 24.4 V as $h_{d}$ decreases from 80 nm to 40 nm. By fixing the value of $h_{d}$ at 50 nm and improving $V_{0}$ from 1 to 5 V, we found that $V_{g}$ can further decrease to 5.9 V, which means that using doped graphene in MGIS is an effective method to create a realistic tunable CRIT effect in our proposed system suitable for practical application.

## 5. Conclusions

In this study, we proposed a novel photonic crystal system, which includes a PCW and a defect cavity side-coupled with a resonant cavity embedded with MGIS. As confirmed by simulations on COMSOL, the resonant wavelength of CRIT in our designed system could be effectively tuned from 1255.1 nm to 1260.9 nm as the chemical potential of MGIS decreased from 0.8 eV to 0.4 eV. Meanwhile, the value of the transmission peak is verified to be closely related to the structural parameters, such as the lattice constant, radius of silicon unit in defect cavity, and so on. In addition, a multipeak CRIT effect was realized in a four-resonator-coupled photonic crystal system. By tuning the chemical potentials of MGISs in different cavities felicitously, we can obtain four-peak CRIT, three-peak CRIT, and two-peak CRIT. What is more, we also found that the voltage required to tune the chemical potential of MGIS from 0.8 eV to 0.4 eV can be effectively reduced to 5.9 V via doping graphene. All these results confirm the excellent characteristics of our proposed system, which provides promise for further research in the field of graphene-based integrated nanophotonic devices, multichannel-selective filters, and optical sensors.
